# Bridging the Digital Divide: Developing Tech Support for Rural Veterans to Improve Telehealth Access

**DOI:** 10.1093/geroni/igaf122.2766

**Published:** 2025-12-31

**Authors:** Elizabeth Marfeo, Elizabeth Chamberlin, Steven Shirk, Victoria Ngo, Maria Venegas, Cathy Cruise, Lauren Moo

**Affiliations:** Tufts University, Medford, Massachusetts, United States; VA Bedford Health Care System, Bedford, Massachusetts, United States; VA Bedford Health Care System, Bedford, Massachusetts, United States; VA Bedford Health Care System, Bedford, Massachusetts, United States; Department of Veterans Affairs, Bedford, Massachusetts, United States; Veterans Health Administration, Northpoint, New York, United States; VA Bedford Healthcare System, Bedford, Massachusetts, United States

## Abstract

Telehealth expansion has the potential to improve healthcare accessibility, yet older rural Veterans continue to face a significant digital divide due to factors such as technology skills and comfort, as well as inadequate telehealth infrastructure. This presentation describes the challenges during the pre-implementation phases of developing the Telemedicine and Coaching for Older Adults to Connect in the Home (T-COACH) program. The T-COACH is an in-home volunteer support-based technology training program to facilitate increased telehealth access for older rural Veterans. This program aims to provide tailored education and training to empower Veterans to access their healthcare appointments via telehealth. By integrating face-to-face in-home education delivered by rurally residing trained community-based volunteers, this initiative offers a scalable approach to bridge the Digital Divide for many older Veterans living in rural areas. However, implementation in rural locales presents unique challenges, including recruiting rural volunteers with technical and interpersonal skills, transportation limitations, multi-tiered training, administrative regulations, and ensuring home-based safety and quality control. Our initial findings suggest successful deployment requires collaboration with local organizations, appropriate resource allocation, and strategic volunteer engagement. Future efforts should focus on refining training frameworks, optimizing volunteer recruitment, and securing sustainable funding models to ensure long-term program viability.

